# Reconsidering Aromatase for Breast Cancer Treatment: New Roles for an Old Target

**DOI:** 10.3390/molecules25225351

**Published:** 2020-11-16

**Authors:** Jessica Caciolla, Alessandra Bisi, Federica Belluti, Angela Rampa, Silvia Gobbi

**Affiliations:** Department of Pharmacy and Biotechnology, Alma Mater Studiorum-University of Bologna, Via Belmeloro 6, I-40126 Bologna, Italy; jessica.caciolla2@unibo.it (J.C.); alessandra.bisi@unibo.it (A.B.); federica.belluti@unibo.it (F.B.); angela.rampa@unibo.it (A.R.)

**Keywords:** aromatase, aromatase inhibitors, allosteric modulation, breast cancer, drug development, multitarget, estrogen receptors

## Abstract

The current therapeutic approach for the treatment of hormone dependent breast cancer includes interference with estrogen receptors via either selective modulators or estrogens deprivation, by preventing their biosynthesis with aromatase inhibitors. Severe side effects and acquired resistance are drawbacks of both drug classes, and the efforts to overcome these issues still allow for research in this field to be animated. This review reports on recent findings that have opened new avenues for reconsidering the role of aromatase enzymes (and estrogen receptors) leading to the possibility of looking at well-known targets in a new perspective.

## 1. Introduction

Despite the increasingly advanced understanding of the molecular biology underlying the onset and progression of breast cancer (BC), this disease still represents the type of cancer that most frequently affects women and one of the leading causes of death among females worldwide [1]. Several risk factors, namely family history and obesity, but also a genetic predisposition due to mutations of tumor-suppressor genes, such as BRCA1 (breast cancer susceptibility gene 1) and BRCA2 (breast cancer susceptibility gene 2), responsible for DNA repair, have been related to higher BC incidence [2]. Notably, while observing an increase in diagnoses, a reduction in mortality can also be noticed, mainly due to early identification and fast therapeutic treatment of BC. However, the occurrence of resistance or disease recurrence renders a long-term therapy even more complex.

In a high percentage of cases, BC has proven to be estrogen dependent, since high levels of these hormones are needed for its growth and proliferation. Therefore, the development of an effective therapy for the treatment of this pathology over the years has been relying on the inhibition of estrogens effects. In this respect, human aromatase (HA), key enzyme for the biosynthesis of estrogens, and estrogen receptors (ERs) have long been thoroughly investigated as primary targets for endocrine therapy, and in particular, recent findings regarding the structure of HA have paved the way for new studies in this field. Indeed, research has now turned the spotlight back on HA, stimulating the interest in this old target from a different point of view. In this review, the latest emerged approaches are reported, which could shed new light on the role of HA in the modulation of estrogens level and lead to the design and development of new drug candidates acting with a different mechanism, allowing the overcoming of intrinsic drawbacks of currently used drugs.

## 2. Estrogens and Estrogens Receptors

**Figure 1:**
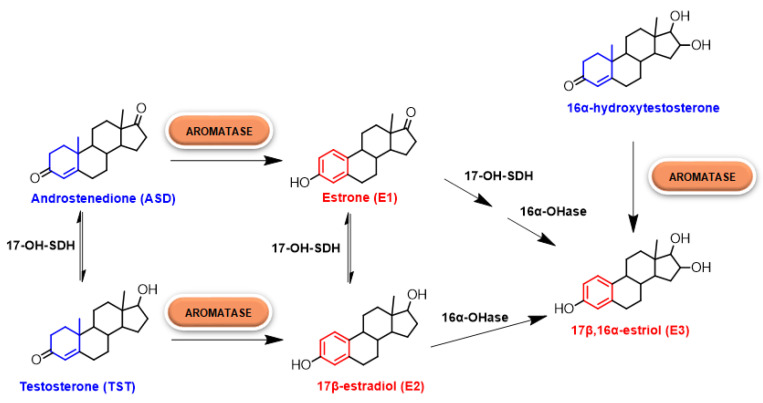
Estrogens’ biosynthesis. In particular, the role of human aromatase (HA) is highlighted.

Estrogens, belonging to the family of steroid hormones, are synthesized starting from cholesterol, transported inside the mitochondria and converted into pregnenolone, the precursor of all steroid hormones, which is then exposed to an extensive metabolism by 17-hydroxilase/17,20-lyase enzyme to give androstenedione (ASD). ASD is then converted into the two most common estrogens in the body, estrone (E1) and estradiol (E2), by the action of two enzymes: 17β-hydroxysteroid dehydrogenase and aromatase (CYP19A1). In particular the latter, belonging to the superfamily of cytochromes P450, converts ASD into E1 and testosterone (TST) into E2 (Figure 1) [3,4].

In premenopausal women, estrogens are mainly synthesized by theca and granulosa cells in the ovaries and, in a minimal amount, in several extragonadal tissues such as mesenchymal cells of adipose tissue, osteoblast and chondrocytes, aortic smooth muscle cells, vascular endothelium and various parts of the brain. In postmenopausal women, ovarian synthesis is drastically reduced and extragonadal tissues become the most important source of circulating estrogens [5].

**Figure 2:**
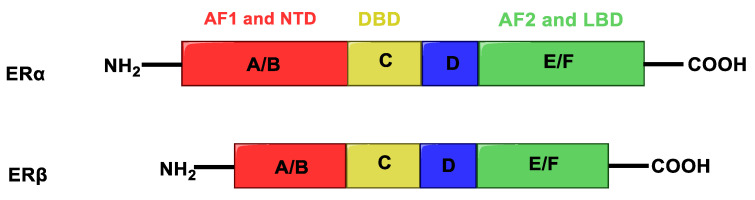
Structure of estrogen receptors. A/B (red) indicates the activation function 1 (AF1) and the N-terminus domain (NTD), C (yellow) the DNA binding domain (DBD), D (blue) the hinge region, E/F (green) the activation function 2 (AF2) and ligand-binding domain (LBD).

Estrogens exert their wide physiological functions by binding to estrogen receptors (ERs), nuclear transcription factors that regulate the mechanism of many physiological functions in humans. ERs exist in two main isoforms, ERα and ERβ, showing high homology, composed of different functional regions: N-terminus domain (NTD), DNA binding domain (DBD) and ligand-binding domain (LBD) (Figure 2).

The transcriptional activity of these receptors is mediated by two different activation functions: AF1, located within the N-terminus domain, that is an independent activation function and shows low similarity between ERα and ERβ, and the ligand-dependent activation domain AF2, located within the LBD. The two receptor subtypes have opposite functions in the cell and a different tissue distribution. ERα is able to stimulate cell growth, and is mainly expressed in ovary (thecal cells), uterus, prostate (stroma), Leydig cells in testis, epididymis, breast and liver; on the other hand, ERβ has an antiproliferative effect, counteracting ERα activities, and is mainly present in prostate (epithelium), testes, ovary (granulose cells), bone marrow and brain [6,7,8].

ERs mediate a complicated signaling network that can be either genomic or non-genomic; moreover, the estrogen-ER complex can bind to the DNA directly or indirectly. The various signaling pathways mediated by ERs can therefore be classified based on their different mechanisms. Direct genomic signaling: binding of estrogen to the receptor leads to a conformational change that allows for the dimerization of the latter. The complex estrogen-ER migrates within the cell nucleus and interacts with chromatin at the level of specific DNA sequences called estrogen response elements (EREs), modulating the transcription of target genes [5]. Indirect genomic signaling: in this case, there is no direct binding between ERs and DNA, but the modulation occurs through protein–protein interactions with the involvement of other transcription factors and their response elements [9]. Non-genomic signaling: estrogens can interact with membrane receptors (some variants of ERα and ERβ and the newly identified G protein-coupled estrogen receptor 1 (GPER1) receptor), causing peculiar changes leading to an indirect modulation of gene expression through the phosphorylation of transcription factors [5,9,10]. Ligand independent signaling: sometimes ERs can be activated in the absence of estrogens or other agonists. This activation is due to the phosphorylation of the receptor at specific residues of serine and tyrosine, mediated by the action of protein kinase A (PKA), protein kinase C (PKC), peptide growth factors, cytokines or neurotransmitters and cell cycle regulators [11,12].

Besides their pivotal role in the regulation of numerous physiological processes, particularly in the development of sexual characteristics and reproductive functions, estrogens also influence other organs’ functioning, such as liver, heart and bone. As previously stated, they also play a key role in BC progression, representing a valuable target for anticancer therapy.

## 3. Aromatase Enzyme

HA catalyzes the conversion of ASD, TST and 16α-hydroxytestosterone to E1, E2 and 17β,16α-estriol (E3), respectively (Figure 1). Its activity is highly specific, being the only enzyme in humans capable of catalyzing the aromatization of androgens to estrogens [13,14].

**Figure 3:**
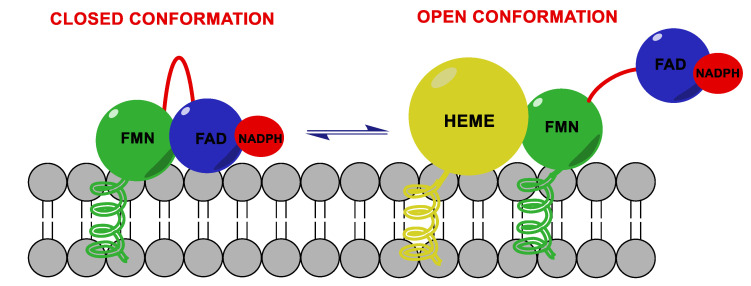
Aromatase complex. Flavin adenine dinucleotide (FAD) and flavin mononucleotide (FMN) domains of NADPH-cytochrome P450 reductase (CPR) can take a “closed” conformation, favoring the internal transfer of electrons, or an “open” conformation, where the two cofactor domains are far away, allowing the transfer of electrons from FMN to monooxygenase.

HA performs its function thanks to the electrons supplied by NADPH-cytochrome P450 reductase (CPR), a transmembrane protein formed by two cofactor domains: the flavin adenine dinucleotide (FAD) domain, which also contains the NADPH binding site, and the flavin mononucleotide (FMN) domain (Figure 3). In addition, a linker domain is situated between the FMN and FAD/NADPH domains. The transfer of electrons is considered to occur from NADPH to FAD and then to FMN. It was observed that CPR can take a “closed” conformation, where the FMN domain is close to FAD/NADPH domain favoring the internal transfer of electrons, or an “open” conformation, where the two cofactor domains are far away. The latter conformation seems to facilitate the intermolecular transfer of electrons from FMN to monooxygenase. The electronic activity of CPR is therefore regulated by the interconversion from the closed to the open conformation [15,16,17].

In 2009, Gosh et al. solved the crystallographic structure of the aromatase-ASD complex [18] for the first time, and this work allowed gaining new insight into the structure of this enzyme, until then hypothesized on the basis of sequence homology with other cytochromes P450 [19,20]. HA is composed of a single polypeptide of 503 amino acids that fold up to form 12 α-helices (A–L) and 10 β-strands (1–10), which are distributed into four sheets, one major and three minor [21]. Considering the crystallographic structure, it was possible to establish that ASD binds to the steroid binding pocket directing its β-face towards the heme group and positioning its C19 4.0 Å from the Fe atom. This specific binding pocket at the active site is formed due to a 3.5 Å distortion of I-helix axis, this displacement being caused by Pro308, a specific residue of HA, absent in the structure of other cytochromes P450. This shift is stabilized by hydrogen bonds between Asp309 and Thr310 (2.8 Å) and between Asp309 and water (3.4 Å) and allows the Asp309 side chain to interact with 3-keto oxygen of ASD [18].

HA converts androgens to estrogens in a three-step process. The first step is a hydroxylation reaction on C19 methyl group producing a 19-hydroxy androgen, which is again hydroxylated in the second step forming a gem diol intermediate [22]. As for the last step, although several studies have been performed trying to understand the details of this reaction and postulating different routes [22,23,24], the underlying mechanism has not been fully elucidated yet.

## 4. Endocrine Therapy for Estrogen-Dependent Breast Cancer

To date, there are two main approaches for the treatment of estrogen positive (ER+) BC, directed at two different targets: the first relies on molecules that act on ERs by selectively modulating their activity, while the second focuses on blocking the endogenous synthesis of estrogens by inhibiting the aromatase enzyme.

### 4.1. Modulation of Estrogen Receptors

**Figure 4:**
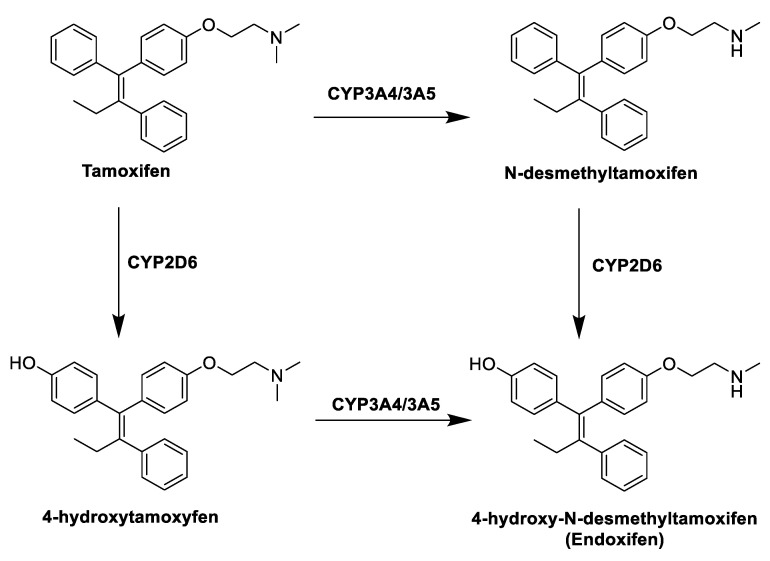
Tamoxifen (TAM) and its metabolites.

The first class of compounds developed for the treatment of ER + BC is represented by SERMs (selective estrogen receptors modulators), non-steroidal compounds binding to ERα and ERβ and endowed with a tissue-selective pharmacology, showing agonist activity in some tissues, such as bone, liver and cardiovascular system, and antagonist activity in other tissues, such as brain and breast, and mixed agonist/antagonist activity in the uterus [25,26]. This specificity is due to several factors, namely, tissue specific expression of the receptors, different availability of co-factors in various tissues and different conformational changes of the receptors induced by ligand binding [27]. Indeed, SERMs compete with estrogens and modulate ERs activity thanks to conformational changes that influence the binding of various cofactors, with which the receptors are associated. When estrogens bind to the hydrophobic pocket of the LBD, the receptor adopts a conformation in which helix 12 can seal the ligand into the ligand-binding pocket, activating AF2 and allowing the binding of cofactors to the receptor. Inversely, when a SERM binds to the LBD, its side chain prevents helix 12 from sealing the binding pocket and the repositioning of the helix hampers the binding of cofactors to AF2, blocking receptor activation [28,29]. The prototype of this class of compounds is tamoxifen (TAM, Figure 4), with a triphenylethylene structure, approved by the Food and Drug Administration (FDA) in 1977 and currently widely used for the treatment of ER + BC in pre- and postmenopausal women. TAM is administered as single *Z*-isomer (as the citrate salt), endowed with higher affinity for estrogens receptors than its *E* counterpart [30].

TAM is in fact a prodrug, and within the body it undergoes extensive metabolism by different cytochromes P450 that convert it into three active metabolites (Figure 4): 4-hydroxytamoxifen, *N*-desmethyltamoxifen and 4-hydroxydesmethyltamoxifen (endoxifen, END). The anticancer effect of TAM is thus mainly due to the activity of these metabolites, in particular 4-hydroxytamoxifen and END, produced by hydroxylation and demethylation of the drug by the action of hepatic CYP3A4/3A5 and CYP2D6 [31]. Despite its therapeutic advantages, the use of TAM is limited by the development of intrinsic or acquired drug resistance [32] and its notable side effects, mostly in a long term therapy, among which the increased risk of developing endometrial cancer, caused by its agonist effect in the uterine tissue. This risk is dose- and time-dependent, and several studies have shown that patients taking TAM have two to three times greater risk of developing endometrial cancer than the rest of the population [33].

**Figure 5:**
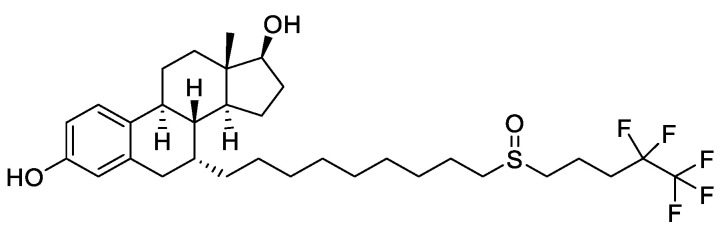
Structure of the selective estrogen receptor degrader (SERD) fulvestrant.

A more recent class of drugs that proved to be highly effective in modulating ERs is represented by selective estrogen receptor degraders (SERDs). The binding of these compounds to ER inhibits the activation of AF1 and AF2 domains, hinders the translocation of the receptor inside the nucleus and causes its degradation. Fulvestrant (Figure 5), a steroidal derivative, is the only SERD currently in use in BC therapy and is able to competitively bind the ER acting as pure antagonist. As it does not act as partial agonist in healthy tissues such as the uterus, its side effects are less pronounced than those of TAM [28,34].

### 4.2. Aromatase Inhibitors

**Figure 6:**
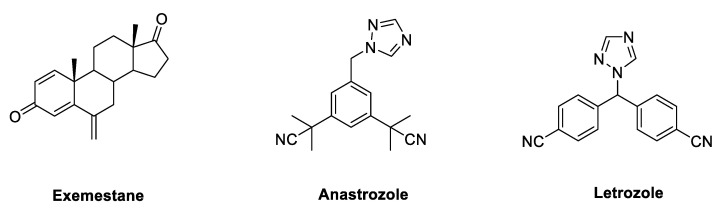
Marketed third generation aromatase inhibitors (AIs). These drugs feature either a steroidal (exemestane) or nonsteroidal (anastrozole and letrozole) structure.

Due to its key role in the synthesis of estrogens, HA has long been regarded as a crucial target, to which small molecules could be directed in the development of endocrine therapy fighting BC. Aromatase inhibitors (AIs) bind to the enzyme and block its activity, inhibiting the endogenous synthesis of estrogens and drastically reducing the circulating levels of these hormones throughout the body. According to their chemical structure and their mechanisms of action, marketed AIs are divided into two classes, namely, steroidal and non-steroidal blockers. Steroidal AIs (exemestane, EXM [35], Figure 6) have a structure deriving from ASD, the natural substrate of HA, and they covalently bind the enzyme causing an irreversible inhibition. Non-steroidal AIs (anastrozole [36] and letrozole, LTZ [37], Figure 6) are derivatives featuring nitrogen-containing heterocycles that establish non-covalent interactions with the heme group of the enzyme by coordinating its Fe atom, resulting in a reversible inhibition [38,39,40,41].

These commercially available compounds, belonging to the third generation of AIs, have high specificity for HA without interfering with the biosynthesis of other steroid hormones and, to date, they are the first line endocrine therapy for the treatment of post-menopausal ER + BC. Nevertheless, the complete depletion of estrogen levels in the whole body caused by inhibition of this enzyme leads to the development of various side effects, such as musculoskeletal pain, reduction of bone density, increase of fractures and cardiovascular events [42,43,44,45]. Several studies have shown that third generation AIs, in particular LTZ, are superior to TAM as first-line therapy for advanced BC. In particular, the BIG (Breast International Group) 1-98 trial compared five years of TAM versus LTZ as monotherapy and the treatment for two years with one of these drugs followed by three years treatment with the other in postmenopausal women with ER-positive BC. It was found that treatment with LTZ as monotherapy led to an improvement in terms of disease-free survival, overall survival, distant recurrence-free interval and BC-free interval, with respect to TAM [46]. The ATAC (Arimidex, tamoxifen, alone or in combination) trial compared five years of anastrozole alone with TAM alone or in combination. Again, it was found that the treatment with AI led to an improvement of disease-free survival and time-to-recurrence compared with TAM. Moreover, treatment with anastrozole reduced the incidence of contralateral BC and of other drug-related adverse effects such as endometrial cancer, thromboembolic events, ischemic cerebrovascular events, hot flushes and vaginal discharge compared with TAM. On the other side, TAM led to a reduction of fractures and arthralgia observed with treatment with anastrozole [47,48]. For their superior clinical efficacy, it is reasonable to consider AIs as the adjuvant endocrine treatment of choice for post-menopausal women with this kind of cancer.

## 5. Emerging Roles for Aromatase Enzyme as BC Target

Despite the highly effective clinical outcome of the currently available therapies for ER + BC, some issues still need to be fully addressed, and keep researchers’ attention focused on both the identification of novel targets involved in the onset of this disease and the optimization of the engagement of the validated ones. Indeed, the complete depletion of estrogens due to the treatment with AIs leads to increased frequency of cardiovascular events and, mainly, reduction of bone density. On the other side, SERMs also present significant side effects, especially in long-term therapies, among which is the increased risk of endometrial cancer. Moreover, the development of intrinsic or acquired drug resistance was also observed for both classes of drugs [32,49]. Thus, considerable efforts are devoted to the development of novel drugs that could help to overcome these issues, and a number of studies have also been carried out to get further insight into the specificities of the related targets. In particular, a significant crosstalk between HA and ERs was recently discovered, and the results of these studies opened new avenues for reconsidering the role of HA enzyme, leading to the potential development of novel classes of small molecules able to interfere in different modes with this well-known target.

### 5.1. Potential Fine Tuning of HA Enzymatic Activity through Allosteric Modulation

It is well known that the ER modulation mechanism of TAM is partly due to the activity of its metabolites, still endowed with high affinity for ERs and potent antiestrogenic activity. In 2012, in an attempt to rationalize the observed biological actions and side effects of the treatment with TAM, which did not appear to be directly correlated to the concentration of its active metabolites, Lu et al. postulated a possible role for these molecules as AIs. Indeed, a significant HA inhibiting activity was demonstrated for some TAM metabolites [50,51], confirming that the interaction with this enzyme can play a major role in the overall clinical effects seen with the treatment with TAM. In an earlier paper [50], a detailed in vitro study was carried out for TAM, END, *N*-desmethyltamoxifen (NDMT) and *Z*-4-hydroxytamoxifen (Figure 4), and a micromolar inhibition of HA was proved for END and NDMT, while no significant effect was observed with TAM or *Z*-4-hydroxytamoxifen. Remarkably, a non-competitive or allosteric inhibition of the enzyme was proposed for the two compounds, allowing postulating potential interactions of small molecules with binding sites different from the catalytic site, which had not been considered before. In a second paper [51], the effects on HA of a large number of TAM metabolites were studied in vitro, and most of them were shown to have activity as AIs, with a wide range of potencies (see later).

Based on the results postulating a non-competitive mechanism of action on HA for TAM metabolites, an in-depth study of the potential location of allosteric binding sites was performed by the group of Magistrato and coworkers [52]. In particular, by applying multiple computational methods, two putative binding pockets in different regions of HA were identified (named Site 1 and Site 2), which can bind TAM metabolites forming stable complexes. Moreover, with the use of molecular dynamics (MD) simulations, the binding of END into one of these pockets was seen to remarkably reduce the flexibility of the enzyme and interfere with a functional collective motion crucial for the breathing of the channel for substrate uptake/release, leading to an impairment of HA catalytic activity. More recently, the same research group tried to get new information on putative entry/exit channels accessible to a substrate (ASD) and a nonsteroidal inhibitor (LTZ) of HA [53]. Using an integrated computational protocol, it was seen that these two molecules, characterized by different size, shape and lipophilicity, could access HA active site by distinct entry routes, while their egress would preferentially follow one channel only. Remarkably, both channels were found to be located close to the putative HA allosteric sites previously identified.

Around the same time, Ghosh and coworkers observed a non-competitive or mixed mechanism for the inhibition of HA when studying the marketed AI LTZ and other azole fungicides [54], presenting the possibility that the biological effect of this drug could be the result of different modes of interaction with the enzyme. In this respect, the position of possible additional binding sites on HA was studied and seemed to be in agreement with the abovementioned results of Magistrato. In particular, besides the androgen-specific catalytic site in the heme distal region, another cavity was found along the entrance channel to HA’s active site, where binding of a ligand could thus hamper the entry of the substrate, while an additional pocket was positioned within the heme proximal region, at the coupling interface with HA redox partner CPR, so that binding to this site may interfere with the necessary electron transfer. Furthermore, binding of a small molecule to these regions may also allosterically cause conformational changes in the active site. The heme proximal region was later further explored by the same authors [55], and postulated to be able to bind either the redox partner or a non-steroidal molecule, being larger than the corresponding area of most cytochromes P450, and that this interaction could influence HA enzymatic activity.

**Figure 7:**
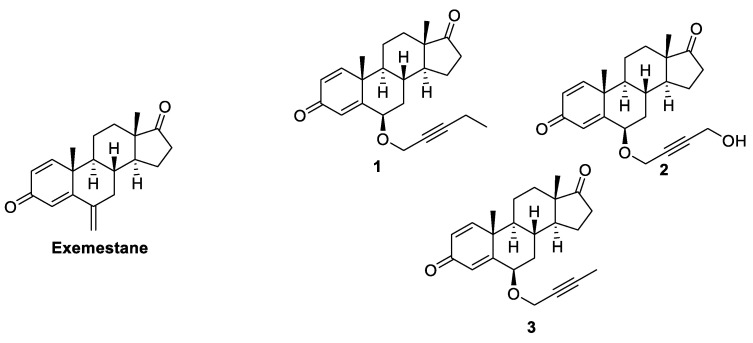
Steroidal HA inhibitors shown to occupy the active site access channel.

These observations raised the possibility that an allosteric mechanism might contribute to the pharmacologic regulation of aromatase and could be exploited to modulate its activity for therapeutic benefit. In fact, already in 2012 the same research group synthesized a series of derivatives of EXM by inserting several C6 *β*-alkynyloxy side chains with the aim of developing new steroidal inhibitors [56]. Analyzing the crystal structures of ASD and EXM complexes with HA, they observed that the steroid skeleton in the binding site was surrounded by hydrophobic residues and by proton donor groups capable of forming H-bonds with 3- and 17-keto oxygens. The only exposed positions of the steroid were C4 and C6, which were near the opening of the active site access channel. Substitutions at these two positions could occupy the available space and, in particular, the insertion of a rigid carbon chain could effectively penetrate the “hydrophobic clamp” (an entry point between Ser478 and Thr310) into the access channel. To confirm these molecular modeling predictions, a series of 6-substituted andro-1,4-diene-3,17-dione containing different alkynyloxy chains was synthesized, and the HA inhibitory and antiproliferative activities of the new compounds were evaluated. It was seen that compounds with a six-atom rigid chain (Figure 7, compounds **1** and **2**) were the most potent inhibitors of HA with IC_50_ values (12 and 20 nM, respectively) comparable to that of the potent non-steroidal AI LTZ (10 nM). Any variation in the length of the chain led to an increase in IC_50_, suggesting that the six-atom chains had the ideal size to fit in the active site cleft. The same result was observed in the antiproliferative assay; these new compounds abolished the TST-stimulated proliferation of MCF-7a cells in a dose dependent manner and the best compound, with EC_50_ of 0.03 nM, was again compound **1**, with the pentynyloxy chain substituent. Furthermore, the X-ray structures of the HA complexes of two new inhibitors (**1** and **3**, Figure 7) were compared with those of ASD and EXM. It was observed that the side chain of the new inhibitors protruded and fit into the access channel cavity, occupying what would later have been postulated to be one of the hypothetical allosteric sites. These observations suggested that the simultaneous occupation of orthosteric and allosteric sites might be a new effective inhibitory strategy.

**Figure 8:**
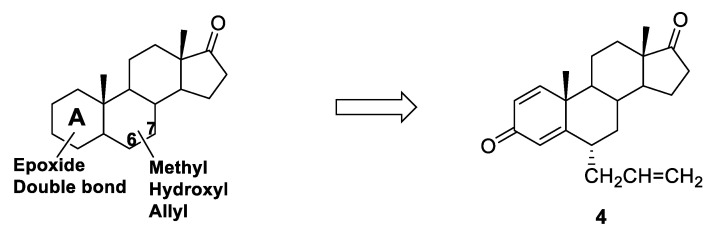
General structure of C6 or C7 substituted steroidal AIs able to fit into HA access channel and most potent compound of the series (4).

Another study evaluating the effect of substitutions in positions 6 and 7 of the steroid core was performed by Roleira et al. [57]. Starting from the hit compounds identified in their previous works [58,59,60] (in which a double bond or epoxide functions were inserted in different positions of ring A), new series of compounds were synthesized (Figure 8) carrying methyl, hydroxyl or allyl groups at C6 or C7 positions of the steroid backbone. C6-substituted compounds generally showed a higher HA inhibitory activity with respect to the corresponding C7 derivatives, and docking analysis showed that the substituent in position 6 was able to better fit into the access channel of HA, and thus to better interact with the enzyme, with respect to the same substituent on C7. From this study, compound **4** (Figure 8) emerged as a potent irreversible AI, showing an IC_50_ (55 nM) in the same range of exemestane.

**Figure 9:**
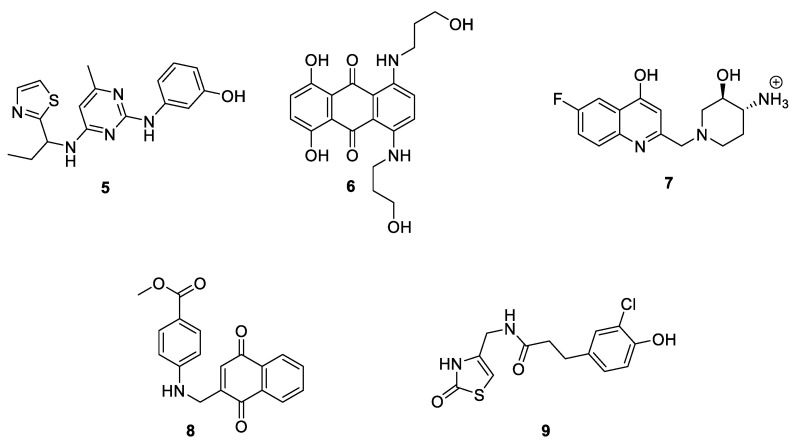
Allosteric modulators of HA identified by virtual screening.

A proof-of-principle study of the feasibility of allosteric modulation of HA was performed in 2019 by Magistrato and coworkers [61]. With the aim to exploit a new therapeutic approach that could reduce side effects and delay the development of acquired resistance of the therapeutic strategies currently in use, an integrated computational and experimental protocol was developed to identify commercially available small molecules able to inhibit HA in a non-competitive or mixed way. Given the recent discovery of the mixed/non-competitive inhibition mechanism of LTZ [54], they initially performed docking of this inhibitor on the two putative allosteric sites investigated in their previous work [52]. It was seen that, by binding to Site 1, LTZ was able to displace the network of water molecules crucial for HA activity as source of protons for its catalytic activity. The same behavior was observed for *E*-END, which could thus hamper proton delivery. Both LTZ and END could also firmly bind into Site 2, the heme proximal cavity indicated to stabilize the interaction between HA and CPR. Then, a virtual screening of different ligand libraries was performed, followed by MD simulation and calculation of the binding free energy (ΔG_b_) that allowed a subsequent refinement in the number of hits. The best molecules were then tested in preliminary inhibitory assays and five molecules (**5**–**9**, Figure 9) showed HA inhibition activity in the µM range. Compounds **5**, **6** and **7** were identified by virtual screening performed on Site 1, while compounds **8** and **9** on Site 2. The antiproliferative activity of these compounds and LTZ on MCF-7 (ER+) and MDA-MB-231 (ER–) cell lines was also evaluated. Except for **7**, the other molecules were able to reduce the growth of MCF-7 cells, while only compounds **6** and **8** showed some antiproliferative activity on ER– cells. Finally, enzymatic kinetics assays confirmed a non-competitive or mixed mechanism of action for the selected compounds.

**Figure 10:**
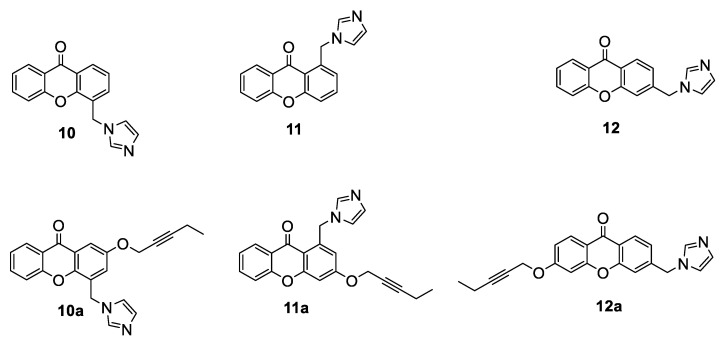
Design of potential nonsteroidal dual-acting AIs.

Based on the abovementioned results and with the aim of developing dual-acting AIs, non-steroidal compounds purposely designed to interact with both the active and the postulated allosteric sites, our research group has recently performed some modifications on a series of imidazolylmethylxanthones (compounds **10**–**12**, Figure 10) [62] that had previously been shown to be potent and selective AIs. In particular, the introduction on the scaffold of a pentynyloxy chain, which had proven to be the most suitable group for interacting with the residues lining the access channel in steroidal compounds [56], was suggested (compounds **10a**–**12a**, Figure 10) [63]. Docking studies of xanthones **10**–**12**, imposing a constraint on Fe-N bond, were first performed and indicated that, for the design of the new derivatives, the best position to insert the pentynyloxy chain in order to fit into the access channel was meta with respect to the imidazole ring. Two compounds (**10a** and **11a**) were thus designed and synthesized. Considering the binding pose predicted for compound **12** using docking calculation with constraint on the Fe-N bond, no favored position for the chain to fit inside the access channel could be found. Thus, for this compound, docking without constraint on the Fe-N bond was also performed, and it was observed that the carbonyl oxygen was close to the heme group and was able to coordinate its Fe atom. Considering this peculiar rearrangement, compound **12a** was also designed and synthesized. Biological evaluation of compounds **10a** and **11a** showed a notable decrease of HA inhibitory potency with respect to parent compounds **10** and **11**, while derivative **12a** maintained an activity similar to **12**. Thus, the functionalization with the alkoxy chain close to the imidazole moiety (as in **10a** and **11a**) seemed to be responsible for hampering the correct positioning of the compounds in the active site, while the same chain located in the opposite ring (**12a**) did not influence the interaction with the target. To give a rational explanation to these biological data, classical MD and quantum mechanism/molecular mechanism (QM/MM) simulations were performed. The results suggested that, for these derivatives, an optimal coordination between the imidazole nitrogen atom and the heme group of the enzyme was not critical to obtain good inhibitory activity, since no stable coordination bond was seen for the most potent **12a**. Moreover, while the pentynyloxy function of the steroidal compounds could perfectly occupy and close the access channel, in compound **10a** the hydrophobic tail could not optimally fit into it, only causing its rearrangement and leaving it partially open. Docking calculation and MD simulation were also performed on the two putative allosteric sites and indicated that only compound **12a** proved to be able to fit in the site near the access channel (Site 1), but spontaneously dissociated after a few nanoseconds of MD stimulation, while all three compounds **10a**–**12a** dissociated after 50 ns of MD simulation after binding to Site 2. The designed compounds can be regarded as useful tools to explore the possibility to target both the allosteric and orthosteric sites of HA.

### 5.2. Multipotent Agents Targeting Both HA and ERs

The multitarget approach has gained more and more importance in the last decades, being considered one of the most potentially effective strategies to tackle complex multifaceted pathologies, due to the advantages of the development of a single molecular entity able to concurrently interfere with multiple interconnected targets. In the field of BC treatment, the potential synergistic activity of the combination of drugs interfering with different pathways involved in this pathology has been, and still is, largely explored, taking into account new promising targets being discovered over the years. As for ER + BC, different studies have been performed in which the two current therapeutic options for endocrine therapy, HA inhibition and ER modulation, were compared and combined in a polypharmacology treatment, as in the aforementioned ATAC trial [47,48], where five years’ therapies with anastrozole or TAM alone were compared with the combination of the two drugs. Moreover, the potential synergistic effect of the simultaneous administration of SERDs and AIs was evaluated by Brodie et al. in a trial in which the therapy with LTZ alone was compared with fulvestrant alone or in combination [64]. Indeed, data showed that, when these two compounds were administered together, the antitumor activity was higher than when they were administered individually. Moreover, with the aim of overcoming the development of resistance observed after a few years of AIs therapy, numerous researchers have focused their attention on several combinations of AIs with molecules that interfere with different mechanisms underlying the development of tumor adaptation. All these studies have been recently reviewed by Daldorff et al. [65].

**Figure 11:**
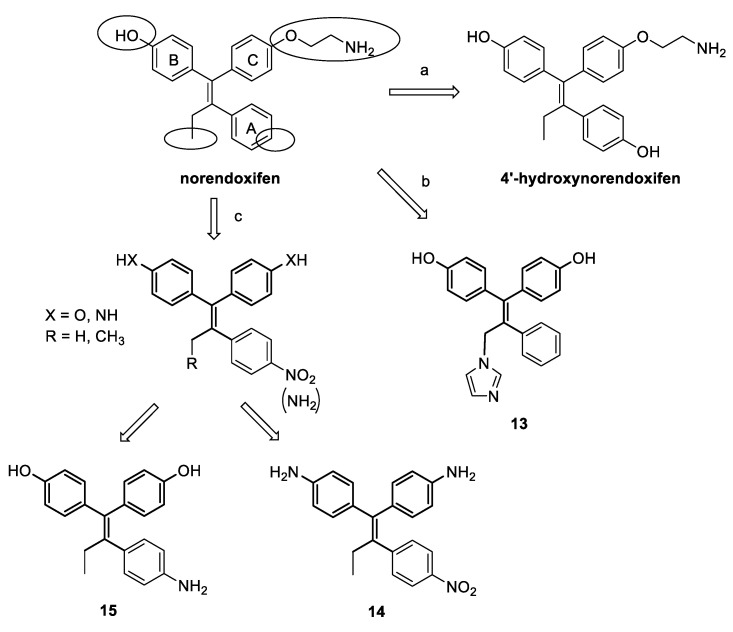
Structure-activity studies on norendoxifen. Main modifications led to the introduction of a hydroxyl group on ring A (a), removal of amino side chain and introduction of heme-coordinating moieties (b) and substitutions with amino or nitro groups (c).

Nevertheless, the feasibility of a multitarget strategy had in fact not been explored until a few years ago. In this respect, the work of Lu and coworkers [51] on TAM metabolites led to the first evidence of single molecules endowed with a significant activity on two targets involved in the development and progression of ER + BC. As mentioned before, the potential HA inhibition activity of a large number of TAM metabolites was explored, and most of them were indeed seen to be competitive AIs, with a wide range of potencies. Here, some interesting SAR remarks could be made, since subsequent hydroxylations and demethylations of the structure of TAM all led to an increase in inhibitory potency. In particular, the demethylated derivative of END (norendoxifen, Figure 11), a minor and previously unrecognized active metabolite of TAM, proved to be the most potent compound, with an IC_50_ value of 90 nM, and was shown to competitively inhibit the enzyme. Docking studies were performed to explain the observed range of activities for the different metabolites, and norendoxifen binding mode was specifically studied. The obtained models allowed postulating that, given the size of the active site of HA in the area of interaction of the amino group of TAM metabolites, the high potency of demethylated derivatives could be due to their lower steric hindrance; or, since the amino group was seen to form a hydrogen bond with the carbonyl oxygen atom of Ala306, the loss of its capacity to act as a hydrogen bond donor may account for the decrease in potency seen with the presence of methyl groups in the amine of the least potent compounds. (*E*,*Z*)-norendoxifen was later synthesized, together with its *E*- and *Z*-isomers, and further studied by Cushman and colleagues [66]. Its activities on both HA and ERs were confirmed and, in particular, (*E*)-norendoxifen proved to be the most potent isomer on HA, while (*Z*)-norendoxifen had slightly higher binding affinity for ERs.

The evidence of the potent HA inhibition activity showed by norendoxifen paved the way for the possibility to purposely design small molecules that could effectively interfere with the activity of the enzyme and simultaneously act as SERMs, so that HA inhibitory activity would stop estrogen biosynthesis in breast tissue, with the consequence of synergistically acting with SERM in inhibiting tumor growth, while ER modulation activity could reduce the side effects caused by complete estrogen depletion in bones and other tissues. Moreover, the structure of this TAM metabolite could represent a new lead scaffold in the search for novel AIs, being markedly different from any previously reported agent, maybe endowed with improved effects and toxicity profiles.

A first series of norendoxifen derivatives was synthetized by the group of Cushman [67], searching for the optimization of activities on both HA and ER. Based on the previously obtained binding models [66] of the lead molecule with HA and ERs, a detailed structure-activity study was performed (Figure 11, route a) by modifying different fragments, namely, the hydroxyl group on ring B and the terminal amino group or the ether oxygen of the side chain on ring C, and by introducing an additional hydroxyl on ring A and different Fe-coordinating (CN, imidazole or 1,2,4-triazole) or alkyl moieties on the ethyl group. From the testing results it appeared that the hydroxyl on ring B and the ethyl moiety seemed to be optimal for activities on both targets, while the introduction of Fe-coordinating groups increased HA inhibition, but remarkably decreased ER binding, and replacement of the amino group in the side chain was generally detrimental for activity. Among the modifications performed, the insertion of a hydroxyl on ring A gave the most promising results. Indeed, 4′-hydroxynorendoxifen (Figure 11) showed increased activities toward HA, ERα and ERβ with respect to the parent norendoxifen, with IC_50_s of 45 nM, 15 nM and 9.5 nM, respectively, as well as higher selectivity.

Another series of derivatives was synthetized by the same research group [68] in order to improve the biological profile and in particular to avoid isomerization, since the different isomers of norendoxifen have proven to have different biological activities against the targets, and interconversion could thus reduce the effects of the drug and facilitate the development of resistance. To this aim, the aminoethoxy side chain was replaced by a hydroxyl group leading to a symmetric bisphenol structure, on which different Fe-coordinating moieties on the ethyl group and hydrogen-bond donors (hydroxyl or amino groups) on ring A were introduced (Figure 11, route b). Testing results indicated the insertion of an imidazole moiety as the most favorable modification for an optimal effect on both HA and ERs, regardless of the presence of substituents on ring A, which generally had only a minor influence or reduced activity. Compound **13** (Figure 11), was shown to significantly inhibit HA and efficiently bind ERα and β, with IC50s of 4.77 nM, 27.3 nM and 40.9 nM, respectively.

In a further attempt to optimize the structure of norendoxifen, some new derivatives were later synthesized, in which a nitro or an amino group were placed in the para position on ring A, and the aminoethyl moiety on ring C was replaced by either a hydroxyl group, to give bisphenols as in previous series, or an amino group that together with a hydroxyl/amino switch on ring B led to a series of bis-aniline derivatives. For all compounds, the ethyl moiety was also shortened to give a corresponding methyl derivative (Figure 11, route c). These modifications were supposed to lead to potent dual agents, even in the absence of the nitrogen-containing side chain or imidazole ring found in previous molecules, through the introduction of other basic nitrogen atoms. While the insertion of the nitro group on ring A was detrimental for activities for bisphenol compounds, their corresponding aniline derivatives showed high potency on both HA and ERα and β, with IC_50_s of 62.2 nM, 72.1 nM and 70.8 nM, respectively, for compound **14** (Figure 11). Reduction of the nitro group on ring A to amino group had diverse effects on the binding affinity to ERs, while it led to remarkably high inhibitory activity on HA for both bisphenols and anilines, providing AIs with low nanomolar potency, comparable to marketed drugs (**15**, IC_50_ = 8.8 nM, Figure 11).

**Figure 12:**
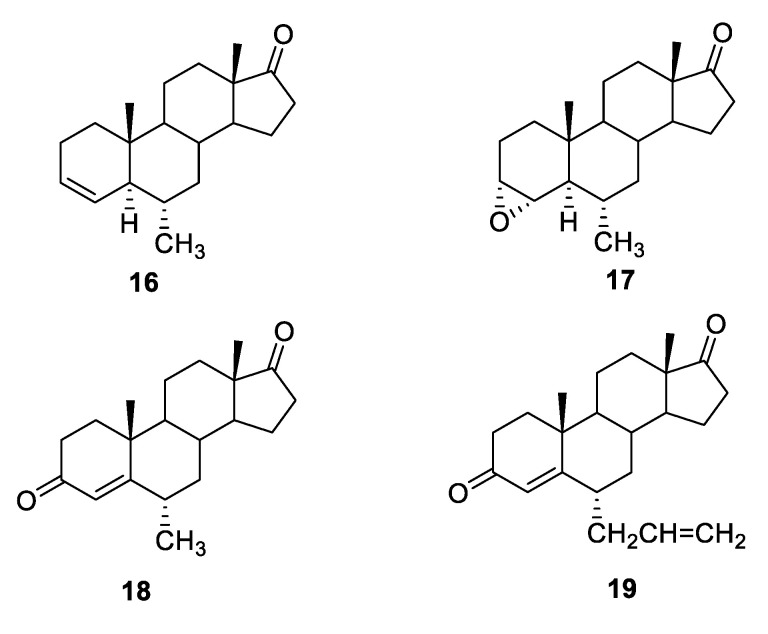
Potential multitarget AI/SERM steroidal compounds.

Recently, the mechanism of the antitumor effects of some of the steroidal AIs earlier described by Roleira [57] was evaluated, and the potential involvement of the modulation of steroid receptors, in particular estrogen and androgen receptors (AR), was investigated [69]. For this purpose, cells were treated with the tested compounds plus the SERD fulvestrant or the AR antagonist casodex (CDX), and it was observed that the activity of some compounds (**16**–**19**, Figure 12) was reduced in these assay conditions. The correlation between the decreased activity of the compounds and the degradation of ERs by fulvestrant or the block of AR by CDX suggested that the action of these derivatives could also be mediated through the interaction with ER or AR.

## 6. Conclusions

Hormonal manipulation still stands as a pivotal therapeutic option and represents a key strategy to face ER + BC. Unfortunately, both AIs and SERMs are endowed with side effects, which can complicate a long-term therapy. Moreover, the onset of drug resistance could make it difficult to obtain a favorable prognosis. These drawbacks could be addressed by discovering new therapeutic targets or finding new potential in old ones. HA enzyme is undergoing renewed interest, thanks to both the hypothesis of a previously unexplored allosteric modulation, and the exploitation of the multitarget approach.

The allosteric modulation of estrogen biosynthesis could offer significant advantages with respect to competitive drugs, since allosteric drugs could achieve the maximal inhibitory potency without a complete inhibition of estrogen production, and their effects would not be reduced at higher concentrations of natural substrates. Moreover, targeting allosteric sites, less conserved than active sites across protein families, may offer the possibility of developing more selective drugs, limiting off-target interactions and side effects.

On the other hand, the multitarget approach may lead to the identification of drugs endowed with an improved efficacy with respect to the widely used combination of drugs, overcoming the pharmacokinetic issues arising from the administration of separate compounds, simultaneously increasing patients’ compliance. The development of a single molecule able to reduce estrogens’ activity by interfering with different steps of the same pathway could also reduce the incidence of side effects usually seen with SERMs or AIs treatment. In this respect, recent studies seem to confirm the possibility of obtaining balanced AI/SERM dual target agents that could represent an effective strategy to tackle this disease.

In view of the results obtained in recent studies, the hybridation of molecules directed to different biological targets involved in the progression of ER + BC could be further exploited by the use of a variety of agents (AIs, SERMs or SERDs) to obtain more effective drugs. Moreover, the latest studies on HA structure and the possibility of the fine tuning of its action via allosteric modulators have allowed for the reconsideration of this key enzyme from a new perspective, and the proof of principle of the feasibility of this approach will lead to a renewed interest for the search and development of new classes of AIs.
